# Genetic Structure and Triazole Antifungal Susceptibilities of Alternaria alternata from Greenhouses in Kunming, China

**DOI:** 10.1128/spectrum.00382-22

**Published:** 2022-05-12

**Authors:** Guangzhu Yang, Sai Cui, Nan Ma, Yuansha Song, Jun Ma, Wenjing Huang, Ying Zhang, Jianping Xu

**Affiliations:** a State Key Laboratory for Conservation and Utilization of Bio-Resources in Yunnan, Key Laboratory for Southwest Microbial Diversity of the Ministry of Education, Yunnan University, Kunming, People’s Republic of China; b School of Life Science, Yunnan University, Kunming, People’s Republic of China; c Horticultural Research Institute, Yunnan Academy of Agricultural Sciences, Kunming, People’s Republic of China; d Department of Biology, McMaster University, Hamilton, Ontario, Canada; Mycology Laboratory, Wadsworth center

**Keywords:** allelic diversity, *cyp51*, genetic differentiation, human fungal pathogen, plant fungal pathogen, population genetics, short tandem repeats, triazole resistance

## Abstract

Alternaria alternata is an opportunistic human fungal pathogen and a ubiquitous phytopathogen capable of causing diseases to >100 agricultural crops and ornamental plants. To control plant diseases caused by A. alternata, triazole fungicides have been widely used both in open crop and vegetable fields and in indoor growth facilities such as greenhouses. At present, the effect of fungicide use on triazole resistance development in A. alternata populations is not known. Here, we isolated 237 A. alternata strains from nine greenhouses around metropolitan Kunming in Yunnan, southwest China, determined their genotypes using 10 short tandem repeat markers, and quantified their susceptibility to four triazoles (difenoconazole, tebuconazole, itraconazole, and voriconazole). Abundant allelic and genotypic diversities were detected among these A. alternata strains. Significantly, over 17% of the strains were resistant to difenoconazole, and both known and new drug-resistance mutations were found in the triazole target gene *cyp51*. Our findings of high-level genetic variation of A. alternata in greenhouses coupled with high-frequency fungicide resistance call for greater attention to continued monitoring and to developing alternative plant fungal disease management strategies in greenhouses.

**IMPORTANCE** Alternaria alternata is among the most common fungi in our environments, such as indoor facilities, the soil, and outdoor air. It can cause diseases in >100 crop and ornamental plants. Furthermore, it can cause human infections. However, our understanding of its genetic diversity and antifungal susceptibility is very limited. Indeed, the critical threshold values for resistance have not been defined for most antifungal drugs in this species. Greenhouses are known to have heavy applications of agricultural fungicides. In this study, we analyzed strains of A. alternata from nine greenhouses near metropolitan Kunming in southwestern China. Our study revealed very high genetic diversity and identified strains with high MIC values against two agricultural and two medical triazole antifungals within each of the nine greenhouses. Our study calls for greater attention to this emerging threat to food security and human health.

## INTRODUCTION

*Alternaria* is a ubiquitous fungal genus distributed in almost every ecological niche across the globe (1). Many members of this genus are opportunistic pathogens capable of causing infections in a variety of crop plants and vegetables (2–4). Some species also cause infections in humans. These fungi typically grow saprophytically, consuming dead organic matter commonly found in soil, but can disperse widely to both outdoor and indoor environments by releasing large numbers of airborne spores. Consequently, they are posing an increasing threat to a diversity of plants, animals, and humans and represent a model group of fungi for the “One Health” framework to study how populations of fungi from the natural environments, agricultural food production, and human health are linked with each other (5, 6).

Among the species in *Alternaria*, Alternaria alternata is probably the best known. This species is a haploid phytopathogen that can cause leaf spot and postharvest rots in >100 agricultural crops and ornamental plants (7). The susceptible host plants include crucifers, eggplants, potato, tomato, cotton, pistachio, apple, citrus, pear, and other economic crops (8–10). In China, diseases caused by A. alternata have been reported on a wide range of plants, including cereal crops, vegetables, and fruits (11–15). Due to the lack of cultivars resistant to this pathogen, diseases such as Alternaria brown spot of citrus, brown spot of tobacco, and early blight of tomato and potato are very common and cause serious economic losses (16, 17). So far, treatments of these plant diseases have relied almost exclusively on applications of fungicides (18). Over the last 20 years, several types of fungicides, including respiratory chain quinone outside inhibitors (QoIs), succinate dehydrogenase inhibitors (SDHIs), and sterol demethylation inhibitors (DMIs), have been approved for treating diseases caused by *Alternaria* spp. for most economic crops in China (http://www.icama.org.cn/hysj/index.jhtml). Among these categories of fungicides, due to their broad spectrum of activity against different fungal pathogens, DMIs such as triazole fungicides difenoconazole (DIF) and tebuconazole (TEB) have been widely used (19). However, resistance to triazole fungicides has been reported (18), reducing the efficacies of these fungicides in controlling plant fungal diseases and posing a substantial threat to agricultural productivity and to public health.

Plant pathogens can mutate and/or adjust their growth pattern, metabolism, and physiology to survive or evade environmental stresses, including fungicides. For example, a recent study revealed that the application of iprodione over 3 years for treating *Alternaria* brown spot of Minneola tangelo led to the development of iprodione resistance in A. alternata (20). Similarly, A. alternata and other *Alternaria* species isolated from pistachio and tangerine orchards became less sensitive to azoxystrobin, pyraclostrobin (QoIs), boscalid, fluopyram, and isopyrazam (SDHIs) after only 3 to 4 years of consecutive applications of these fungicides (21–24). In addition, Avenot et al. found that the sensitivity of A. alternata from pistachio with and without triazole-exposure history (TEB, DIF, and propiconazole) had unimodal but different distribution patterns, suggesting potentially common mechanisms of resistance (10, 23). In contrast, analysis of DIF resistance in A. alternata from seven potato-growing regions in China revealed that A. alternata had a skewed distribution toward higher tolerance (25), suggesting a stepwise accumulation of mutations making them increasingly tolerant to DIF (18). At present, the potential molecular mechanisms of triazole resistance are largely unknown for most drug-resistant A. alternata strains. Understanding the patterns of triazole susceptibility and the potential underlying mechanisms could help develop better strategies to manage fungicide usages against A. alternata.

Triazole fungicides such as DIF and TEB function by inhibiting the sterol 14α-demethylase, an essential enzyme for ergosterol biosynthesis encoded by one or more of the *cyp51* genes (26–28). Previous studies have shown that triazole resistance mechanisms can be grouped into three major types. The first type includes point mutations causing amino acid substitutions in the triazole target gene *cyp51*. For example, mutations at G54, L98, Y121, G138, P214, P216, F219, M220, A284, Y431, G432, G434, and G448 sites of *cyp51A* gene have shown to be associated with DMI resistance in *Aspergillus* (29–35). The second type includes mutations at the promoter region of *cyp51*, leading to overexpression of the triazole target sterol demethylase gene. Several mutations have been reported for the promoter region, including tandem duplications of sequences such as TR34, TR46, and TR53 repeats, that are known to be associated with triazole resistance in *Aspergillus*. Similarly, insertions of 199 bp, 533 bp, and 1,000 bp in the *cyp51* promoter region have been associated with DMI tolerance in Venturia inaequalis, Mycosphaerella graminicola, and Fusarium fujikuroi (26, 36, 37). The third type of mutation includes those that lead to overexpressions of efflux pump-encoding genes such as *abcC* in triazole resistance in *Aspergillus* (34, 38–40). In A. alternata, R511W substitution in *cyp51* was detected in one DIF-resistant isolate, and a 6-bp insertion in the upstream region was observed in half of the DIF-resistant strains isolated from tomato (18). However, little is known about the prevalence of triazole resistance of A. alternata in greenhouses and the potential molecular mechanisms for such resistance. Given the high-intensive usage of greenhouses for agriculture, we expect that A. alternata from greenhouse will show triazole resistance.

An understanding of genetic diversity at the population level is important for inferring the origins and spread of antimicrobial resistance genes and genotypes (41). Such knowledge will also be beneficial for us to develop adequate and effective methods of disease management (42). A variety of molecular markers have been used to analyze the genetic diversity of A. alternata populations from different geographic regions, including isozyme variability, restriction fragment length polymorphism (RFLP), random amplified polymorphic DNA (RAPD), and polymorphic microsatellite markers or short tandem repeats (STRs) (43–48). These studies have found variable levels of genetic variation and evidence of recombination within and among populations of A. alternata, despite that no sexual reproductive structure has been observed for A. alternata from any environment. Meng et al. suggested that the dominant asexual reproduction interspersed with infrequent sexual reproduction contributes to the broad ecological and geographic distribution (49).

In a recent study, we analyzed Aspergillus fumigatus isolates from nine greenhouses in Jinning county, metropolitan Kunming, Southwest China. A high level of genetic diversity and a high frequency of triazole resistance were observed in the greenhouse populations of As. fumigatus (50). Similar to *As. fumigatus*, A. alternata is also a soil-dwelling fungus and is capable of dispersal by airborne asexual spores. However, there are two major differences between these two fungi. First, *As. fumigatus* is among the most common opportunistic human fungal pathogens, while A. alternata is a relatively low-risk opportunistic pathogen for humans. Second, A. alternata can cause diverse plant diseases, while *As. fumigatus* is not known to cause plant disease. In this study, aside from understanding A. alternata in a novel ecological niche (greenhouses) and geographic region (southwestern China), we are also interested in whether populations of A. alternata from the same greenhouses as *As. fumigatus* would have similar patterns of genetic diversity and triazole susceptibility. To achieve our objectives, we isolated A. alternata strains from the same soil samples in the same nine greenhouses in Jinning county, Kunming, China. These isolates were genotyped with a panel of 10 short tandem repeat (STR or microsatellite) markers. In addition, we investigated the susceptibility of these A. alternata isolates to four triazoles, two agricultural triazole fungicides, and two medical triazole drugs, to identify the prevalence of triazole resistance among our A. alternata samples. Finally, the DNA sequences of the triazole target gene *cyp51* were analyzed to identify potential mutations associated with triazole resistance in our strains.

## RESULTS

### Isolation and genetic analysis of A. alternata based on 10 STR markers.

We obtained a total of 237 A. alternata isolates from the 900 soil samples that we originally used to isolate *As. fumigatus* in a previous study (50). Among them, 24, 27, 25, 22, 28, 31, 26, 23, and 31 isolates were obtained from the nine greenhouse populations (pop.1 to pop.9), respectively. The internal transcribed spacer (ITS) sequences of all isolates showed that these isolates all belonged to A. alternata *sensu lato* (see Fig. S1 in the supplemental material). To further confirm the species status, we obtained the DNA sequences for nine randomly selected isolates at the following six gene loci: glyceraldehyde-3-phosphate dehydrogenase (*gapdh*), RNA polymerase second largest subunit (*rpb2*), translation elongation factor 1-alpha (*tef1*), Alternaria major allergen gene (*Alt a 1*), endopolygalacturonase (*endoPG*), and an anonymous gene region (OPA10-2) as suggested by Woudenberg et al. (51). The Bayesian analyses based on ITS sequences and the sequences at the six loci, along with representative species within A. alternata *sensu lato*, confirmed that all nine isolates belonged to A. alternata (Fig. S2).

At each of the 10 STR loci, our analyses revealed that each isolate contained one allele, consistent with all our isolates of A. alternata being haploids. Genotype data based on the 10 STR markers were used to infer genetic diversity and population structure of A. alternata from the nine greenhouses. The results showed that the polymorphic information content (PIC) of each marker was ≥0.5 (Table 1), consistent with all markers used in this study being moderately to highly polymorphic. Two loci, c10524 and PAS2, had high PIC values of 0.84 and 0.83, respectively (Table 1).

**TABLE 1:** Characteristics of the 10 STR loci used for genotyping strains of Alternaria alternata in this study

Locus name	Forward primer sequence (5′–3′)	Reverse primer sequence (5′–3′)	Repeat unit	No. of alleles	PIC^*a*^	Major allele frequency	Gene diversity
c10062	CCTTCTGCTACCTCGGTCTG	GACCTTCTTCCTTGATGCCA	(AC)n	10	0.59	0.3776	0.63
c9473	CGCTCAACCACAGTAACGTC	CTCATCCTCGTCGCTGTCTT	(ACG)n	7	0.59	0.3695	0.63
c10524	TTTCCCTCCTTTCCCTCCTA	CGGGATCTTGATCCGTAGAA	(AG)n	17	0.84	0.1442	0.85
c9860	CGTGATGTCCTGGGATTCTT	GGTGGGCCATCTACTCGTAA	(GTC)n	6	0.6	0.3533	0.64
c3806	GCCTCTTGTAGATTGGCGAG	GTACCCAGGAATGGTAGCGA	(AAG)n	15	0.57	0.3979	0.6
c10756	CAGAAATAGGATGGCGGGTA	TTTTGGCTTTCGAGCACTTT	(CT)n	8	0.7	0.2606	0.74
AEM6 DQ272485	TGACGAGCTGTGAGGAGTGT	HCGTGTGTAGGGTCTTCGTCTC	(CA)n(CT)n	14	0.75	0.2204	0.78
AEM9 DQ272486	GAAGCCCATTCCACTCACA	HGCTCCATCTCCCACAGTAACA	(CAA)n	8	0.51	0.4189	0.58
PAS2	CAATCGTGATGTCGTTACGG	TCGCGCACTGTCTCTCTCTA	(GTC)n	12	0.83	0.1485	0.85
PAS6	CACTTCCCTACGCAGGTAGC	TCGTCTCGCAATTACTCGTG	(CA)n	6	0.5	0.4366	0.56

a PIC, polymorphism information content.

A total of 103 alleles were found at the 10 STR markers, with a mean of 10.3 alleles per marker and a range of 6 (loci c9860 and PAS6) to 17 (locus c10524) per locus in our sample of 237 isolates (Table 1). However, the alleles had uneven frequencies. Sixty of the 103 total alleles had frequencies of less than 5% each, 13 alleles had frequencies between 5% and 10%, and the remaining 30 alleles had frequencies higher than 10%. In the total sample of 237 isolates, the frequency of the most common allele at each locus ranged from 0.22 (PAS2) to 0.60 (C3806 and PAS6) (Table 1). When analyzing allele distributions within each local population, 28 alleles were found in only one greenhouse each, while the remaining 75 alleles were shared by at least two of the nine greenhouse populations (Table 2). Overall, the nine greenhouse populations differed in their total number of alleles and number of private alleles. Briefly, the largest number of alleles was found in pop.9 (61 alleles) and the smallest number of alleles was found in pop.8. Similarly, though not identical to the above, the largest number of private alleles was found in pop.9 (6 private alleles) and the smallest number of private alleles was found in pop.6 and pop.8 (1 private allele in each) (Table 2). The gene diversity of the 10 loci in the total sample ranged from 0.56 (PAS6) to 0.85 (c10524 and PAS2), with a mean value of 0.69.

**TABLE 2:** STR allele distributions and genetic diversity within and among the nine greenhouse populations of Alternaria alternata for each of the 10 STR loci

Population	No. of genotypes	Genetic diversity	No. of alleles at each locus (no. of private alleles in parentheses)										
			c10062	c9473	c10524	c9860	c3806	c10756	AEM6 DQ272485	AEM9 DQ272486	PAS2	PAS6	Total
pop.1	16	0.67	8 (1)	6	6	2	5	4	9	4	7 (1)	7	58 (2)
pop.2	19	0.59	6	4	9 (1)	4 (1)	8	6	6	4	5	4	56 (2)
pop.3	15	0.64	7 (1)	6	8	2	5	5	8 (2)	2	6	2	51 (3)
pop.4	20	0.65	4	6	7 (1)	4	6 (2)	4 (1)	6	4	8 (1)	3	52 (5)
pop.5	25	0.56	4	4	8 (2)	5	7 (1)	4	6	6 (1)	7	4	55 (4)
pop.6	27	0.6	4	5	7	4	4 (1)	5	7	3	7	4	50 (1)
pop.7	26	0.62	4	5 (1)	9 (2)	5	5	6	8 (1)	4	8	4	58 (4)
pop.8	13	0.37	3	3	6	5	5 (1)	3	4	3	6	2	40 (1)
pop.9	26	0.7	7	4	9 (1)	4	6	5 (1)	8	5 (1)	8 (1)	5 (2)	61 (6)
Total	187	0.6	10	7	17	6	15	8	14	8	12	6	103 (28)

Among the 237 A. alternata isolates from the nine greenhouses, we found 187 multilocus genotypes (MLGs) based on the 10 STR loci (Table 2). Of the 187 MLGs, 25 were shared by two or more greenhouses and the other 162 MLGs were found in only one greenhouse population each. The highest number of MLGs was observed in pop.6 (27 MLGs), and the lowest number of MLGs was observed in pop.8 (13 MLGs) (Table 2).

**FIG 1:**
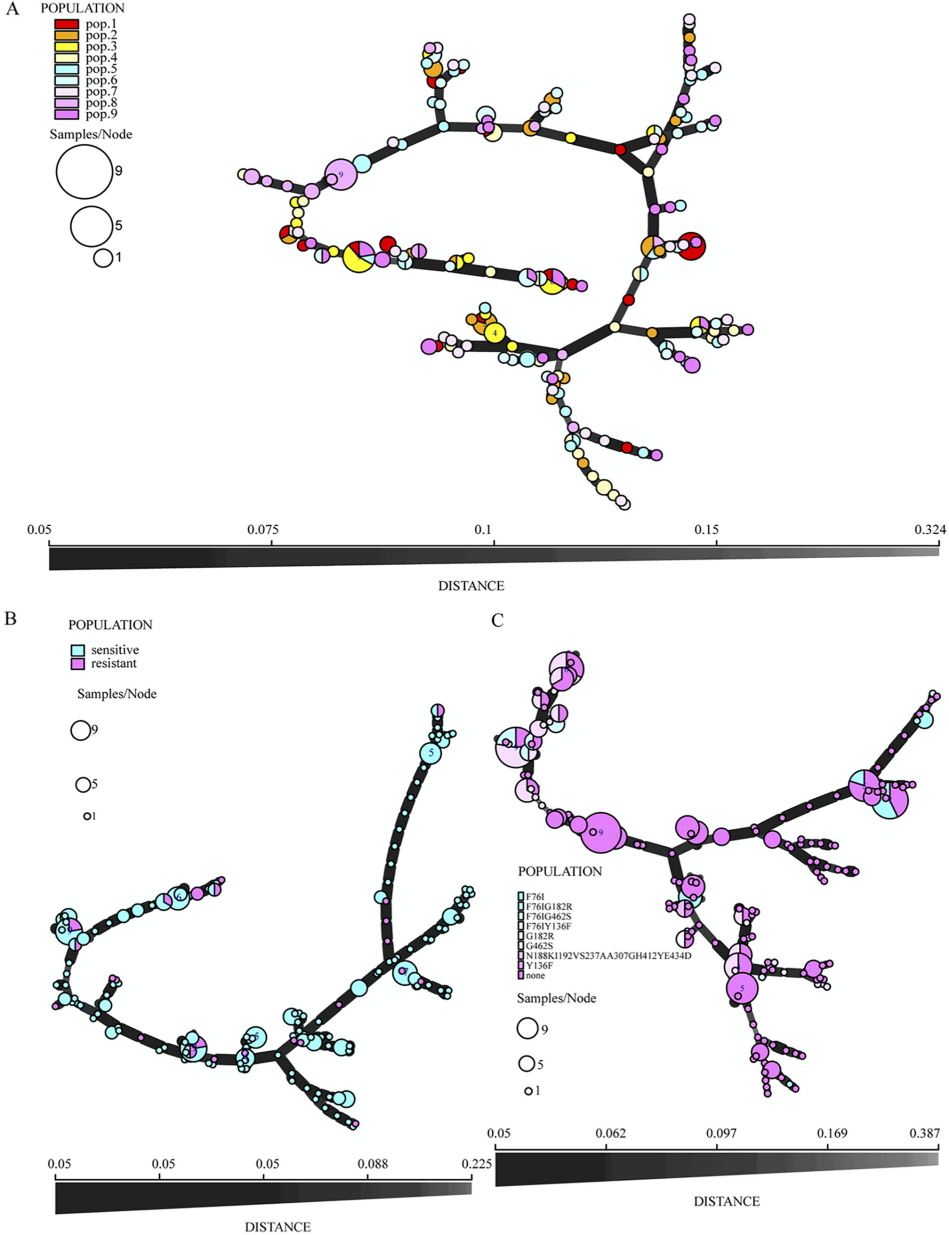
Minimum spanning network (MSN) tree showing the relationships among multilocus genotypes (MLGs) and various strain characteristics. (A) MLG distributions along the MSN tree highlighted according to their greenhouse origins, (B) distribution of difenoconazole resistant isolates, and (C) distribution of amino acid substitutions in *cyp51* gene.

To better visualize (i) the relationships among strains from the nine greenhouses, (ii) the distributions of triazole-susceptible and -resistant strains, and (iii) the distributions of azole resistance-associated mutations in the *cyp51* gene, we first constructed the genotype relationships among all 237 A. alternata isolates based on their Bruvo’s distance between STR genotypes through minimum spanning network (MSN) tree (Fig. 1). Then, the MLGs’ greenhouse location triazole resistance-related mutations at *cyp51* gene, and antifungal susceptibilities were superimposed on the MSN tree (Fig. 1). The results showed that most genotypes from the nine greenhouses were intermixed, with 25 MLGs shared by two or more greenhouses (Fig. 1A). Furthermore, the triazole-resistant strains and triazole resistance-related mutations were widespread across the MSN tree into many STR genotype groups, consistent with multiple origins of resistant strains and resistance-related mutations (Fig. 1B and C).

**FIG 2:**
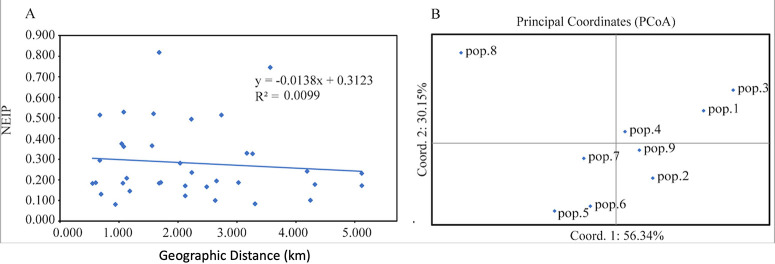
Result of Mantel test and principle-coordinate analysis. (A) Mantel test of the relationship between Nei’s genetic distance (NeiP) for microsatellite markers and geographical distance (GGD) among the nine greenhouse populations of A. alternata. (B) Principle-coordinate analysis (PCoA) based on pairwise population genetic distances.

The analysis of molecular variance (AMOVA) based on clone-corrected data revealed that 96% of the total genetic variation was found within individual greenhouse populations, and a low (4.5%) but statistically significant genetic differentiation was found among the nine greenhouse populations (PhiPT = 0.045, *P = *0.001) (Table S1). We further investigated the extent of genetic differentiation between pairs of greenhouse populations. Our results showed that moderately to highly significant differentiations (*P* < 0.01) were found between pop.8 and all other eight greenhouse populations (Table S2). The biggest differentiation was found between pop.3 and pop.8 (F_ST_ = 0.199, *P = *0.001), followed by that between pop.1 and pop.8 (F_ST_ = 0.182, *P = *0.001) (Table S2). However, Mantel test showed no statistically significant correlation between geographical distances and population genetic distances (correlation coefficient = 0.0099, *P = *0.36; Fig. 2A).

**FIG 3:**
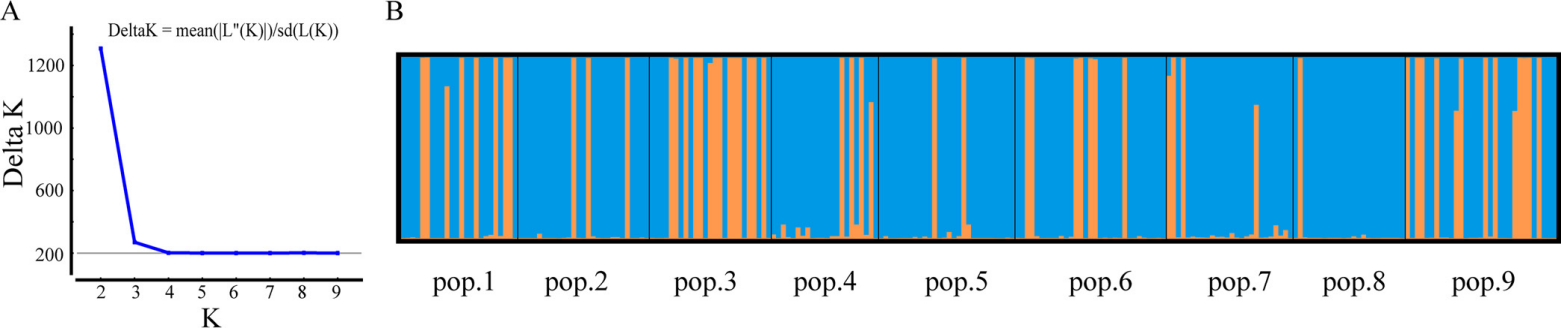
Genetic clusters and their distributions among nine greenhouses obtained from the destruct analysis. (A) Plot of K against delta K. (B) Estimated assignments of strains from each greenhouse to one of two genetic clusters.

Limited but unambiguous evidence for recombination among the 10 STR loci was found in the total sample of A. alternata (PrC = 0, *P = *1; rBarD = 0.187, *P* < 0.001). STRUCTURE analyses revealed that the optimal number of genetic clusters in the total sample was 2 (Fig. 3A). Interestingly, most greenhouses contained strains from both genetic clusters, including a number of strains with genetic elements belonging to both clusters (Fig. 3B). Overall, based on Bruvo’s distance and STRUCTURE results, one cluster contained 140 (59.1%) strains and the other contained 97 (40.9) strains (Fig. S3).

**FIG 4:**
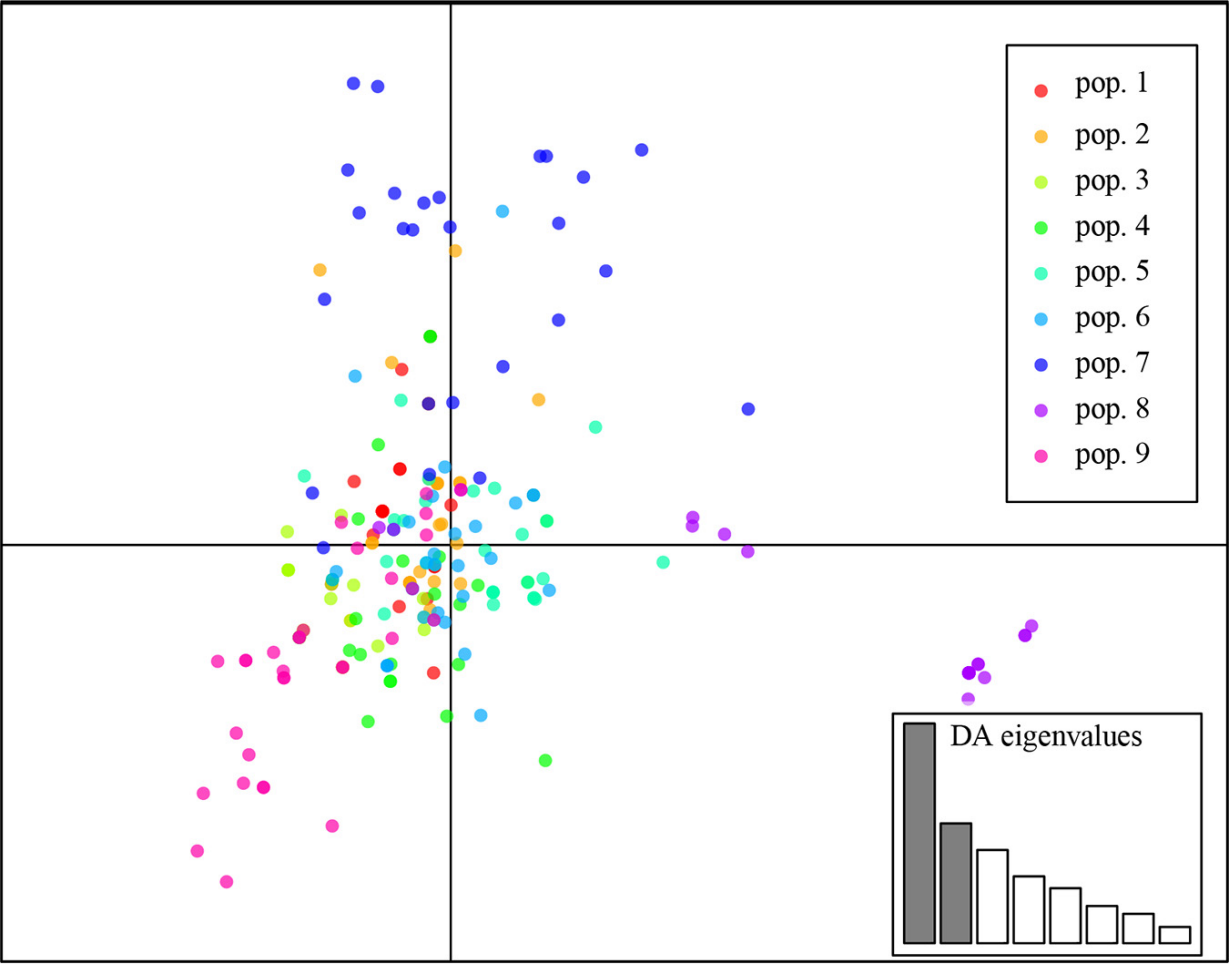
Discriminant analysis of principal components (DAPC) among strains of A. alternata from nine greenhouse populations.

Principle-coordinate analysis (PCoA) based on the pairwise mean population haploid genetic distance revealed that the first and second principal coordinate axes explained 56.3% and 30.2% of the total variation, respectively (Fig. 2B). As shown in Fig. 2B, pop.8 and pop.3 were distinctly different from other greenhouse populations. Furthermore, discriminant analysis of principal components (DAPC) of all populations showed that axis 1 separated pop.8 from the other eight populations (Fig. 4), which is consistent with the result from PCoA and genetic differentiation between pairs of greenhouse populations.

### Susceptibility of A. alternata isolates to triazoles.

A total of 183 isolates representing the nine populations were assayed for their sensitivity to two medical triazole drugs (VOR and ITR) and two agricultural triazole fungicides (DIF and TEB) using a serial dilution of drug concentrations from 16.00 μg/mL to 0.0078 μg/mL in a microtiter plate format. The results showed that the MIC values of the 183 isolates to DIF, ITR, and TEB ranged from ≤0.0078 μg/mL to ≥16.00 μg/mL. The MIC_50_ values of 183 isolates to DIF, ITR, and TEB were 1 μg/mL, 1 μg/mL, and 8 μg/mL, respectively. However, the lowest MIC to VOR was higher than those of the other three triazoles, and the range of MICs for VOR was from 0.125 to ≥16.00 μg/mL (MIC_50_ = 4 μg/mL) (Table 3). Less than 20% of the isolates had MICs of <0.125 μg/mL to any of the four triazoles, with the specific percentages being 4.4% (8/183), 16.9% (31/183), and 7.7% (14/183) for MIC of <0.125 μg/mL to TEB, DIF, and ITR, respectively. Most of the isolates, 44.3% (81/183), 65.6% (120/183), 72.7% (133/183), and 66.1% (121/183), had MIC values of 0.125 to 4 μg/mL to TEB, DIF, VOR, and ITR, respectively. Except to TEB, less than 30% of the isolates had a higher MIC value of ≥8.00 μg/mL to the three remaining triazoles. Isolates from within the same greenhouse often had highly variable sensitivities to the tested triazoles. For example, 85% (17/20) and 75% (15/20) in pop.3 had MIC values of ≥8.00 μg/mL to TEB and VOR, but none of the isolates had the same MIC value to DIF and ITR. Overall, high MICs to DIF, VOR, and ITR were common within each of the nine greenhouses, with 50% (11/22) in pop.9, 75% (15/20) in pop.3, and 68.18% (15/22) in pop.8 showing MIC values of ≥8.00 μg/mL for DIF, VOR, and ITR, respectively.

**TABLE 3:** Distributions of triazole susceptibilities among Alternaria alternata samples from nine greenhouses in Kunming, Yunnan, China

Pop	No. of isolates	Proportion of strains (no. of isolates within MIC value range/total no. of isolates) within each MIC range of the following four triazoles:										
		TEB			DIF			VOR		ITR		
		<0.125 μg/mL	0.125–4 μg/mL	≥8 μg/mL	<0.125 μg/mL	0.125–4 μg/mL	≥8 μg/mL	0.125–4 μg/mL	≥8 μg/mL	<0.125 μg/mL	0.125–4 μg/mL	≥8 μg/mL
pop.1	20	20	40	40	50	20	30	85	15	15	85	0
pop.2	22	18.2	45.5	36.4	31.82	68.2	0	68.2	31.8	0	72.7	27.3
pop.3	20	0	15	85	5	95	0	25	75	0	100	0
pop.4	17	0	47.1	52.9	5.9	82.4	11.8	100	0	5.9	88.2	5.9
pop.5	14	0	50	50	21.4	71.4	7.1	100	0	28.8	57.1	14.3
pop.6	24	0	33.3	66.7	4.2	87.5	8.3	79.2	20.8	20.8	33.3	45.8
pop.7	22	0	63.3	36.4	27.3	54.6	18.2	81.8	18.2	4.6	77.3	18.2
pop.8	22	0	59.1	40.9	4.6	68.2	27.3	63.6	36.4	0	31.8	68.2
pop.9	22	0	45.5	54.6	4.6	45.5	50	63.6	36.4	0	59.1	40.9
total	183	4.4	44.3	51.4	16.9	65.6	17.5	72.7	27.3	7.7	66.1	26.2

### Cross-resistance among the four tested triazoles.

Due to the lack of consensus definition for defining triazole resistance in A. alternata, we investigated potential cross-resistance among the four triazoles using the Pearson correlation analysis based on their MIC values. Specifically, a statistically significant positive correlation in MIC values between two triazoles would signify evidence for cross-resistance in our A. alternata sample to the two tested triazoles. Our results revealed significant positive correlations between MIC values of DIF and VOR (*r* = 0.22, *P = *0.003), DIF and ITR (*r* = 0.47, *P = *0), VOR and TEB (*r* = 0.59, *P = *0), and VOR and ITR (*r* = 0.18, *P = *0.016) (Table 4). Together, these results indicate prevalent cross-resistance in this sample and that a high MIC for one triazole is often associated with a high MIC for a different triazole. However, instances of a strain with a high MIC for one triazole but a low MIC for a different triazole were also observed, suggesting that some of the triazole resistance mutations were unique to a specific triazole drug.

**TABLE 4:** Pearson correlation coefficients between gene diversities and the frequencies of triazole resistance among greenhouse populations of Aspergillus fumigatus and Alternaria alternata^*a*^

Pearson correlation coefficient of *As. fumigatus*:	Pearson correlation coefficient of A. alternata:				
	Gene diversity	Resistance frequency to triazole^*b*^			
		ITR	VOR	TEB	DIF
Gene diversity		−0.54	−0.446	0.245	0.221
ITR resistance	−0.012		0.178*	0.084	0.467**
VOR resistance	−0.47	−0.184**		0.589**	0.216**
TEB resistance	−0.474	−0.177**	0.144*		0.105
DIF resistance	NT^*c*^	NT	NT	NT	

a The susceptibility of Aspergillus fumigatus strains from these greenhouses to DIF was not determined in the study by Zhou et al. (50).

b *, *P* < 0.05; **, *P* < 0.01.

c NT, not tested.

### Triazole fungicide residues in soil samples and relations to azole susceptibilities.

The soil samples used for isolating A. alternata were the same as those used for isolating *As. fumigatus* in our previous study (50). The residues of the two triazole fungicides (TEB and DIF) for these nine greenhouses have been reported previously (50). The concentrations of TEB and DIF ranged from 0.00251 mg/kg to 0.34515 mg/kg and 0.00328 mg/kg to 0.14293 mg/kg, respectively (Table S3) (50). Statistical analysis showed that the concentrations of TEB and DIF residues in the soil of each greenhouse were not correlated with the MIC_50_ value of each greenhouse population of A. alternata or the overall genetic diversity based on STR (Table S4).

### Comparative analysis of *cyp51* gene and upstream sequence.

A total of 68 single nucleotide polymorphisms (SNPs) and 69 haplotypes were observed among the 224 isolates in which we successfully amplified the *cyp51* gene. Among the 68 SNPs, 56 and 12 were in exons and introns, respectively. Of the 56 SNPs in the exons, 43 were synonymous and 13 were nonsynonymous. Most of the synonymous sites (25/43, 58%) were observed in fewer than three isolates each, while 30% (13/43) of the synonymous SNPs, including 120C→G, 180C→T, 370C→T, 466C→T, 469C→T, 575C→T, 1226C→T, 1352C→T, 1361G→A, 1364T→C, 1433T→C, 1482G→A, and 1484T→C, were detected in every greenhouse population with overall frequencies of about 24% (53/224), 26% (58/224), 28% (63/224), 35% (79/224), 28% (63/224), 29% (64/224), 17% (38/224), 17% (38/224), 17% (38/224), 67% (150/224), 7% (16/224), 29% (64/224), and 68% (152/224) in the total sample, respectively.

The 13 nonsynonymous SNPs resulted in 11 amino acid substitutions, including F76I, Y136F, G182R, N188K, V192I, S237A, A307G, H412Y, E434D, G448S, and G462S (Table 5). Among these mutations, only one isolate had the G448S mutation and two isolates had the Y136F mutation. Three isolates had a combination of three substitutions, A307G, H412Y, and E434D. However, 6 of the 224 isolates had six mutations in each (N188K, V192I, S237A, A307, H412Y, and E434D). Except for the amino acid substitution G462S, which was widespread in every greenhouse population, the remaining shared mutation types were each observed in two to seven of the greenhouses (Table 5).

**TABLE 5:** Distribution of amino acid substitutions within the *cyp51* gene among the nine greenhouse populations of Alternaria alternata

Mutation site	Substitution type	No. of isolates with the amino acid substitution in each greenhouse										Frequency (%, of 224 isolates)
		pop.1	pop.2	pop.3	pop.4	pop.5	pop.6	pop.7	pop.8	pop.9	Total	
76	F→I	13	14	0	0	0	0	0	8	0	35	15.63
136	Y→F	1	0	1	0	0	0	0	0	0	2	0.45
182	G→R	1	0	0	0	0	0	0	0	2	3	1.34
188	N→K	2	1	0	1	1	0	0	1	0	6	2.68
192	V→I	2	1	0	1	1	0	0	1	0	6	2.68
237	S→A	2	1	0	1	1	0	0	1	0	6	2.68
307	A→G	2	1	0	1	1	0	1	1	0	7	3.13
412	H→Y	2	1	0	1	1	0	1	1	0	7	3.13
434	E→D	2	1	0	1	1	0	1	1	0	7	3.13
448	G→S	0	0	0	0	1	0	0	0	0	1	0.45
462	G→S	5	5	17	3	4	8	9	2	11	65	28.57

In order to reveal the polymorphism of promoter region of *cyp51* gene, the 1,921-bp upstream sequence of *cyp51* gene was amplified using primers Ucyp-F/Ucyp-R. An insertion of 6 bp located at position −31 to −36 was observed with a frequency of 66% (148/224) in the total sample. One additional nucleotide insertion at position −339 was detected in 29% (64/224) of the isolates. Interestingly, 63 of 64 isolates with insertion at −339 position simultaneously possessed the 6 bp insertion described above.

The chi-square goodness-of-fit test (χ^2^ test) was used to evaluate the association between the observed mutations and the frequency of isolates in different MIC ranges. The results found that amino acid substitutions of F76I (*P = *0) and G462S (*P = *0.005) and the 6-bp insertion (*P = *0.014) in the upstream region of *cyp51* gene were significantly associated with the frequencies of A. alternata isolates distributed in MIC < 0.125 μg/mL, 0.125 μg/mL ≤ MIC ≤ 8 μg/mL, and MIC ≥ 16 μg/mL to DIF. The amino acid substitution of G462S (*P = *0) and the 6-bp insertion (*P = *0.01) were significantly associated with the frequency of isolates in the same three MIC ranges to TEB. In addition, significant associations were detected between F76I (*P = *0), G462S (*P = *0), and the 6-bp (*P = *0.024) insertion and the frequencies of A. alternata isolates distributed in MIC ≤ 1 μg/mL, 2 μg/mL ≤ MIC ≤ 4 μg/mL, and MIC ≥ 8 μg/mL to VOR. However, for the same MIC ranges to ITR, no statistically significant association was detected in F76I, G462S, or the 6-bp insertion genotype groups.

### Comparisons with *As. fumigatus* populations from the same greenhouses.

To explore the potential broader mechanisms affecting fungal population structures in greenhouses, we compared our observations on the A. alternata population genetic patterns with those of *As. fumigatus* populations from these nine greenhouses. Specifically, we conducted a series of Pearson correlation tests based on data from these two filamentous fungi. Our analysis revealed that the gene diversities of these two species were negatively correlated (Table 6 and Table S5), suggesting some form of potential competition between these two species. However, the correlation was not statistically significant. To determine the potential correlation between gene diversity and triazole resistance within and between these two species, we arbitrary defined triazole resistance of the two species at the same MIC for each drug (Table 6). Our analyses revealed negative correlation between genetic diversity and the frequency of triazole resistance in *As. fumigatus* for all four triazoles (Table 4), consistent with drug selection reducing genetic diversity in *As. fumigatus*. However, only two of the four triazole drug-resistance levels showed negative correlation to genetic diversity for A. alternata, while the remaining two showed positive correlations (Table 4). Though interesting, none of the correlations between genetic diversity and triazole resistance in either species were statistically significant (Table 4). Similarly, for each of the individual triazoles, no statistically significant correlations were observed between the two species in their triazole resistance levels. Together, these results suggested that there were both shared and distinct population structures and triazole resistance patterns between these two filamentous fungi from among the nine greenhouses.

**TABLE 6:** Comparisons between Aspergillus fumigatus and Alternaria alternata from nine greenhouses in their gene diversity and frequencies of triazole resistance

Greenhouse	Mean unbiased gene diversity of:		Frequency of triazole resistance					
			ITR (MIC ≥ 4 μg/mL)		VOR (MIC ≥ 4 μg/mL)		TEB (MIC ≥ 8 μg/mL)	
	*As. fumigatus*	A. alternata	*As. fumigatus*	A. alternata	*As. fumigatus*	A. alternata	*As. fumigatus*	A. alternata
pop.1	0.89	0.695	100	0	85.7	15	89.3	40
pop.2	0.881	0.613	76.9	27.3	0	40.9	11.5	52.2
pop.3	0.881	0.666	33.3	5	3.7	80	21.4	85
pop.4	0.893	0.678	57.1	11.8	7.4	29.4	7.4	50
pop.5	0.836	0.580	50	14.3	25	21.4	33.3	50
pop.6	0.858	0.609	96.6	66.7	31	54.2	58.6	66.7
pop.7	0.904	0.641	95	18.2	15	40.9	30	36.4
pop.8	0.89	0.389	96	95.5	32	95.5	48	40.9
pop.9	0.816	0.721	100	77.3	92.9	72.7	96.4	54.5

## DISCUSSION

For decades, molecular markers have helped assess genetic variations within and among fungal populations (52–54). For *Alternaria* spp., several types of molecular markers have been developed to analyze a diversity of taxonomic, ecological, and epidemiological questions (55–57). So far, markers such as RAPD, AFLP, and RFLP have revealed generally low levels of genetic variation within most populations of A. alternata and that populations of A. alternata from different plant hosts and geographical regions are shown to be generally highly homogeneous. However, such observations were likely due to the low variability and/or ambiguous nature of the markers used in those analyses. Because of their high rate of mutation, codominance, and ease of identification from genomic sequences, short tandem repeats (STRs) have emerged as the preferred markers for population genetic studies, especially for recently evolved populations. Indeed, studies based on STRs have shown patterns of genetic variation and population structure of *Alternaria* spp. different from those based on RAPD, AFLP, and RFLP markers (45, 49, 58, 59). In this study, we analyzed allelic and genotypic diversities of 237 A. alternata isolates collected from nine greenhouses using 10 STR loci. Overall, the observed genetic diversity from these nine greenhouses was higher than that previously reported for an A. alternata population (273 isolates) that included geographically diverse samples from Fujian (southeastern China), Henan (central China), Heilongjiang (northeastern China), and Yunnan (southwestern China) provinces based on seven simple sequence repeat (SSR) markers and for an Alternaria tenuissima population (191 isolates) from four broad regions in China (northeastern, northern, eastern, and northwestern China) (49).

In general, sexual reproduction increases genetic diversity and, in the process, can facilitate natural selection by (i) combining deleterious alleles at different loci from different parents into the same progeny so that such alleles are eliminated more effectively and (ii) combining beneficial alleles from different parents so that the alleles are selected for more effectively (60, 61). In this study, we found high levels of genetic diversity and evidence for recombination within each of the nine greenhouses as well as in the total sample. Indeed, the Shannon’s diversity (I = 1.20) for the total greenhouse populations in the present study was higher than those reported by Yang et al. and Meng et al. (15, 59, 61). Our evidence for recombination in natural populations of this species is consistent with that reported for several *Alternaria* species, including those with or without a known sexual cycle (49, 56, 61, 62).

As expected, due to the close geographic distances among the nine greenhouses, the overall level of genetic differentiation among them was low, at less than 5%. A similar low-level genetic differentiation (~2%) was found among the nine greenhouse populations of another fungus, *As. fumigatus* (50). Both species are capable of producing abundant asexual spores that could be dispersed among greenhouses by wind or by human activities, contributing to their overall genetic similarities. Evidence for gene flow among greenhouses is supported by genotype sharing among greenhouses and by the presence of strains belonging to two different genetic clusters within each of the nine greenhouses. However, though the differentiation is low, several pairs of greenhouses showed statistically significant differences in their gene frequencies. The observed genetic differentiations were consistent with some barriers against random gene flow among the greenhouses. The physical barriers surrounding the greenhouses, coupled with potentially different selective pressures and/or genetic drifts within individual greenhouses, could cause the observed differences in allele and genotype frequencies among these greenhouse populations of A. alternata.

In this study, two genetically distinct clusters of A. alternata were found in our total sample of 237 isolates. The number of two genetic clusters was smaller than those reported previously for *Alternaria tenuissima* and A. alternata based on different population samples (15, 59). In both species in previous studies, the population samples were from several regions across China, with the 191 strains of *A. tenuissima* grouped into four genetic clusters and the 275 A. alternata isolates grouped into three genetic clusters (15, 59). At present, due to the different sets of STR markers used between our study and the two previous studies, the relationships between our genetic clusters in A. alternata and those in previous studies are unknown. However, given the high allelic and genotypic diversity in the small geographic area that we sampled for this study, it is highly likely that southwestern China contains abundant indigenous genetic diversity of A. alternata not present in other parts of China or in other parts of the world.

Interestingly, though statistically insignificant, a negative correlation was observed in gene diversity between A. alternata and *As. fumigatus* among the nine greenhouses. This result suggests that these two species may be competitors in the greenhouse environment. In these greenhouses, both species are prevalent in the soil (as shown by our high isolation rates from the soil for both species) and both live primarily as saprotrophs. As a result, they likely compete for the same nutrients where the competitiveness of one species could have negative consequences on the other. A high genetic diversity in one species could signify that the founding population of the species was diverse, had a high mutation rate, and/or experienced frequent sexual reproduction. In a stable environment, such a population could exert selective pressure against its competitor species where only highly competitive genotypes (thus a low genetic diversity) could be found in the population. To test this hypothesis, the interaction patterns of the strains and genotypes of these two species from these greenhouses need to be determined.

However, different from *As. fumigatus*, A. alternata is an opportunistic fungal pathogen and is the culprit of destructive foliar diseases, such as early blight and brown blotch, in over 100 plants. The additional niche on host plants for A. alternata could provide potentially novel opportunities for selection and evolution to occur for A. alternata that might be absent for *As. fumigatus*. Specifically, to prevent and control early blight and brown blotch in economic and horticultural crops both within and outside greenhouses, many classes of fungicides have been used. Frequent application of these protectant fungicides could have facilitated the emergence of fungicide resistance in these pathogens on crop plants (25). Some of the fungicides applied to plants could end up in the greenhouse soil. Similarly, the evolved drug-resistant genotypes in/on crop plants could also fall to the soil. The identification of a high prevalence of strains from soil with high MICs to fungicides is consistent with the effects of fungicide selection on these two species. In our study, the TEB MIC_50_ of A. alternata strains was overall similar to those of A. alternata from potato fields (10, 11) and pistachio orchards (10, 63), suggesting potentially similar selection pressure by TEB across different planting environments. However, while previous researchers suggested that the risk of developing resistance to difenoconazole in A. alternata was low (25, 64), we found 17% of our isolates showing high difenoconazole MIC values (≥8 μg/mL), higher than the 10% (16/160) reported by a previous study (18). The result suggested that close attention should be paid to difenoconazole resistance and to the effectiveness of this fungicide in managing plant fungal diseases, especially in greenhouse environments. The high frequency of triazole resistance in *As. fumigatus* is also consistent with greenhouses as a hot spot for the development of fungicide resistance.

Interestingly, DNA sequence analyses of the triazole target gene *cyp51A* revealed that both A. alternata and *As. fumigatus* showed multiple origins of resistance to the tested fungicides (65). The diversity of observed mutations at the *cyp51*A gene in triazole-resistant strains of both fungi is consistent with ecological niche heterogeneity within and among the greenhouses. This is especially true for A. alternata, a plant pathogen capable of infecting different crops within these greenhouses, with potentially different pathogen genotypes specializing in different crops. The relatively insulated environment within each greenhouse could also contribute to maintaining the evolved drug-resistance mutations in these greenhouses (25).

A previous study found that the range of MIC values to TEB among strains of A. alternata from pistachio orchard was wider than that to DIF (10). However, we found that the ranges of MICs to TEB and DIF were similar in our greenhouse population of A. alternata. Cross-resistance to fungicides with different modes of action has been reported between fungicides mancozeb and DIF (64). Here, our results showed evidence of cross-resistance between certain triazoles but not others. Specifically, positive correlations were observed between MIC values of DIF and ITR, DIF and VOR, and TEB and VOR. The results suggested that the application of DIF likely selected for both ITR and VOR resistance, while the application of TEB likely selected only for VOR resistance in A. alternata. A similar positive correlation was observed between MICs of TEB and VOR in the greenhouse populations of *As. fumigatu*s. However, the MIC values between ITR and VOR, and between TEB and ITR, were negatively correlated in *As. fumigatus* from these greenhouses. Together, these results suggested that different agricultural fungicides likely selected for different resistance mechanisms and that some of these mutations may make them more susceptible to other drugs. Allelic swaps and/or genetic crosses are needed in order to determine the quantitative contributions of each of these observed mutations to triazole susceptibilities (66, 67).

Interestingly, we found negative correlations between gene diversity and MIC values for both ITR and VOR in our greenhouse population of A. alternata. This result is similar to that found for *As. fumigatus* where MICs of *As. fumigatu*s to ITR, VOR, and TEB were negatively correlated with gene diversity. Together, these results suggest that overall, stronger fungicide pressures likely caused the elimination of more alleles and genotypes in the population, leading to a greater reduction in gene diversity in corresponding greenhouse populations of both species. However, though statistically insignificant, slight positive correlations were observed between gene diversity and the MIC values of TEB and DIF among the nine greenhouse populations of A. alternata. The positive correlations indicate that the two agricultural fungicides may have contributed to the maintenance of genetic variation within these greenhouses. Understanding the specific resistance mechanisms among our isolates for each of the two fungicides TEB and DIF could help reveal the underlying population processes for the observed positive correlations. We hypothesize that resistance mutations against TEB and DIF originated from different genetic backgrounds of the strains of A. alternata within these greenhouses. Many of these mutations offer fitness benefits similar to those of strains of A. alternata, which enabled multiple genotypes to be maintained within each greenhouse. Ecological niche heterogeneity within greenhouses (such as in the soil and on plants) could also contribute to the maintenance of genetic diversity.

Aside from being a saprophyte and a plant pathogen, A. alternata is also recognized as a pathogen of immunocompromised patients and those with significant underlying disease conditions (68). Although infection by *Alternaria* species is typically nonlethal, such infections can reduce mobility and increase burden on the health care system. The is especially the case if the infections were caused by drug-resistant pathogens. In *As. fumigatus*, many clinical triazole-resistant strains of *As. fumigatus* have been proposed as likely originated from environmental sources, especially agricultural environments, that can be spread to distant locations (69). Whether a similar pattern could happen in A. alternata remains to be investigated.

Several mechanisms for triazole resistance have been reported in fungal pathogens, including amino acid substitutions in the drug target gene *cyp51A*, overexpression of the target enzyme CYP51, and overexpression of efflux pumps to reduce intracellular drug concentrations (70–72). Overexpression of the target genes is related mostly to transposon insertion or duplication of DNA fragments in the promoter region or the upstream regulatory region. In our study, we found the same triazole resistance-associated insertion sequence as reported previously (18) in 66% of the A. alternata isolates. Furthermore, we reported a novel 1-bp insertion in the upstream region of the *cyp51A* gene. Triazole resistance caused by a single mutation or by multiple mutations in *cyp51A* has been widely reported (70, 71). Here, we identified a total of 11 amino acid substitutions in our A. alternata population. Among of them, Y136F has been reported in fungicide-resistant strains of several phytopathogens, such as Blumeria graminis and Erysiphe necator (73, 74). However, other amino acid substitutions, including two with high frequencies, F76I and G462S, are reported for the first time here (Table 5). The observed associations between genetic changes and frequency distribution in different MIC ranges in our sample suggest that the two novel substitutions F76I and G462S, the previously reported 6-bp insertion, and the new 1-bp insertion all likely contributed to susceptibility differences among strains to DIF, TEB, ITR, and/or VOR. Additional investigations using targeted mutagenesis, allele swaps, and/or genetic crosses are needed in order to determine their specific contributions to differences in triazole susceptibilities in A. alternata (e.g., 18, 19, 40, 42, 67, 68). Furthermore, whole-genome sequencing and genome-wide association studies of populations of strains with different MICs could help identify additional mutations outside the *cyp51* gene that are associated with triazole susceptibilities in A. alternata (66, 67).

## MATERIALS AND METHODS

### Soil samples, strain isolation, DNA extraction, and molecular identification.

In December 2019, 900 soil samples were collected from nine greenhouses, with 100 soil samples from each greenhouse. These greenhouses were used for the production of fast-growing vegetables, including coriander, Cucurbita pepo, pea, lettuce, and fennel. More detailed information about locations where soil samples were collected for isolating A. alternata were described previously by Zhou et al. (50). To isolate A. alternata from each soil sample, ~0.1 g of soil was suspended in 1.5 mL sterile water and well mixed under 180 rpm for 30 min. Subsequently, 1 mL of this suspension was transferred to a petri dish and mixed with 10 mL of ~55°C potato carrot agar (PCA; 10 g carrot, 10 g potato, and 20 g agar per L medium) supplemented with 2% manganese chloride tetra hydrate (MnCl_2_·4H_2_O) and 50 mg/L chloramphenicol (75). After solidification, the plates were incubated in the dark at 28°C for 5 days. Mold colonies typical of *Alternaria* were confirmed microscopically, and asexual spores were picked using the tip of a very thin needle and transferred to new PCA plates. One isolate was obtained from each plate to minimize isolating strains of the same genotype and phenotype.

Genomic DNA was extracted from the mycelia collected from single spore cultures growing on peptone-dextrose agar (PDA) medium using the cetyltrimethylammonium bromide (CTAB) procedure (76). All isolates were checked morphologically with a light microscope and molecularly by PCR amplification and sequencing of the internal transcribed spacer (ITS) regions. In addition, we obtained DNA sequences at six loci (*gapdh*, *tef1*, *rpb2*, *Alta1*, *endoPG*, and OPA10-2) for nine randomly selected isolates for their species confirmation, based on methods described previously (7, 51).

### STR genotyping.

A total of 39 primer pairs for STR markers previously developed for *Alternaria* spp. (45, 49, 58, 59) were used to screen for PCR amplification success and for fragment length polymorphisms using a subset of our isolates. From the initial screening, we selected 10 STR markers that showed high amplification success and high-level polymorphisms to genotype the entire strain collection from the nine greenhouses. These 10 primer pairs were previously developed for *A. tenuissima* (59), A. alternata (45), and Alternaria solani (49) (Table 1). For STR genotyping, the forward primers were labeled with different fluorescent dyes at the 5′ end. PCR amplifications were performed in a total reaction volume of 25 μL. Each PCR rection included 1 μL DNA template (100 μg L^−1^), 9.5 μL sterile distilled water, 12.5 μL of 2× T5 TSE101 PCR mix (TSINGKE Biotechnology Co. Ltd.), 1.0 μL forward primer, and 1.0 μL reverse primer. The amplification was conducted in an Eppendorf MastercyclerR using the following protocol: an initial denaturation step at 95°C for 5 min, followed by 35 cycles of denaturation at 94°C for 30 s, annealing at 57°C for 30 s, and extension at 72°C for 30 s, and it ended with a final extension for 5 min at 72°C. The lengths of the obtained amplicons were then determined using an ABI 3730 DNA sequencer (Applied Biosystems), with fragments of different lengths at each locus representing different alleles. The allelic combination at the 10 STR loci represented the multilocus genotype for each strain.

### Population genetic analyses.

Different indices of allelic and genotypic diversities were calculated to reveal the polymorphisms of microsatellite markers at the population level. Locus-based diversity indices, including major allele frequency (MAF) and polymorphic information content (PIC), were computed using PowerMarker v3.25 software (77).

The STR genotype data were imported into GenAlEx 6.5 (78) to calculate the pairwise PhiPT values between greenhouse populations of A. alternata as well as to infer the relationships between genetic and geographical distances (Mantel test) among populations. The same program was employed to calculate population genetic diversity indices over all loci, including observed number of alleles (Na), effective number of alleles (Ne), Shannon’s information statistic (I), allelic frequency, number of private alleles (NPA), Nei’s gene diversity (h), and percentage of polymorphic loci (PPL) of A. alternata in individual greenhouses. Analysis of molecular variance (AMOVA), pairwise population genetic distances, and gene flow were inferred using the GenAlEx 6.5, as well.

The overall population differentiation (G_ST_) and gene flow [Nm; Nm = 0.5(1 − G_ST_)/(G_ST_)] were estimated using POPGENE version 1.32 (79). The number of genetic populations within the total sample was determined using STRUCTURE version 2.3.5 with a Bayesian model-based clustering algorithm (80). To estimate the optimal number of genetic clusters in the sample (K), we used a burn-in period of 10,000 in each run, with data collected every 100,000 Markov chain Monte Carlo (MCMC) generations for 10 replications at each K value, with K values ranging from 1 to 10. The optimal K value was inferred using the website STRUCTURE HARVESTER version 0.6.94 (81). Finally, a minimum spanning network tree was generated based on Bruvo’s distance, which was calculated in poppr package in R 4.1 (82).

### Susceptibility testing of A. alternata isolates to four triazoles.

DIF and TEB are the most frequently used triazole fungicides in agricultural fields in China, including those for growing vegetables in greenhouses. Similarly, itraconazole (ITR) and voriconazole (VOR) are commonly used for treating patients with mycotic infections, including aspergillosis. DIF and TEB were purchased from Shanghai Yuanye Bio-technology Co., Ltd. (Shanghai, China), at 97% and 99% active ingredient concentrations, respectively. ITR and VOR were purchased from Shanghai Macklin Biochemical Co., Ltd. (Shanghai, China) at 98% and 99% active ingredient concentrations, respectively. All four triazoles were dissolved in dimethyl sulfoxide, respectively, to obtain stock solution of 1.6 × 10^3^ μg mL ^−1^. All stock solutions were stored at 4°C before use.

Susceptibilities of 183 randomly selected strains representing all populations were determined using a modified CLSI M38-A2 broth microdilution method (83, 84). Briefly, for the broth microdilution test, the stock fungicide solutions were adjusted to 32 μg/mL. Subsequently, 16.00 μg/mL fungicide solution was prepared by adding 100 μL of 32 μg/mL fungicide solutions to 100 μL RPMI 1640 medium with glutamine without bicarbonate (Sigma-Aldrich, St. Louis, MO, USA). The medium was buffered to pH 7 with 0.165 M 3-N-morpholinepropanesulfonic acid and filtered using a 0.22-μm membrane filter. Then, the fungicide agent was serially diluted by 2-fold each from 16.00 μg/mL to 0.0078 μg/mL in the 96-well cell culture plate.

To induce conidial formation for triazole susceptibility testing, all isolates were grown on potato carrot agar for 5 days at 30°C. Approximately 5 mL of sterile 0.85% saline with Tween 20 was placed on top of the sporulating colonies to prepare a suspension by gently probing the colonies with the tip of a transfer pipette. The optical densities of the conidial suspensions at 530 nm were determined and adjusted to between 0.25 and 0.3. Aliquots of 100 μL of each adjusted conidial suspension were added to the medium with serially diluted fungicides in the 96-well plate. The 96-well plates were incubated at 30°C for 72 h before being checked for growth under a microscope.

**FIG 5:**
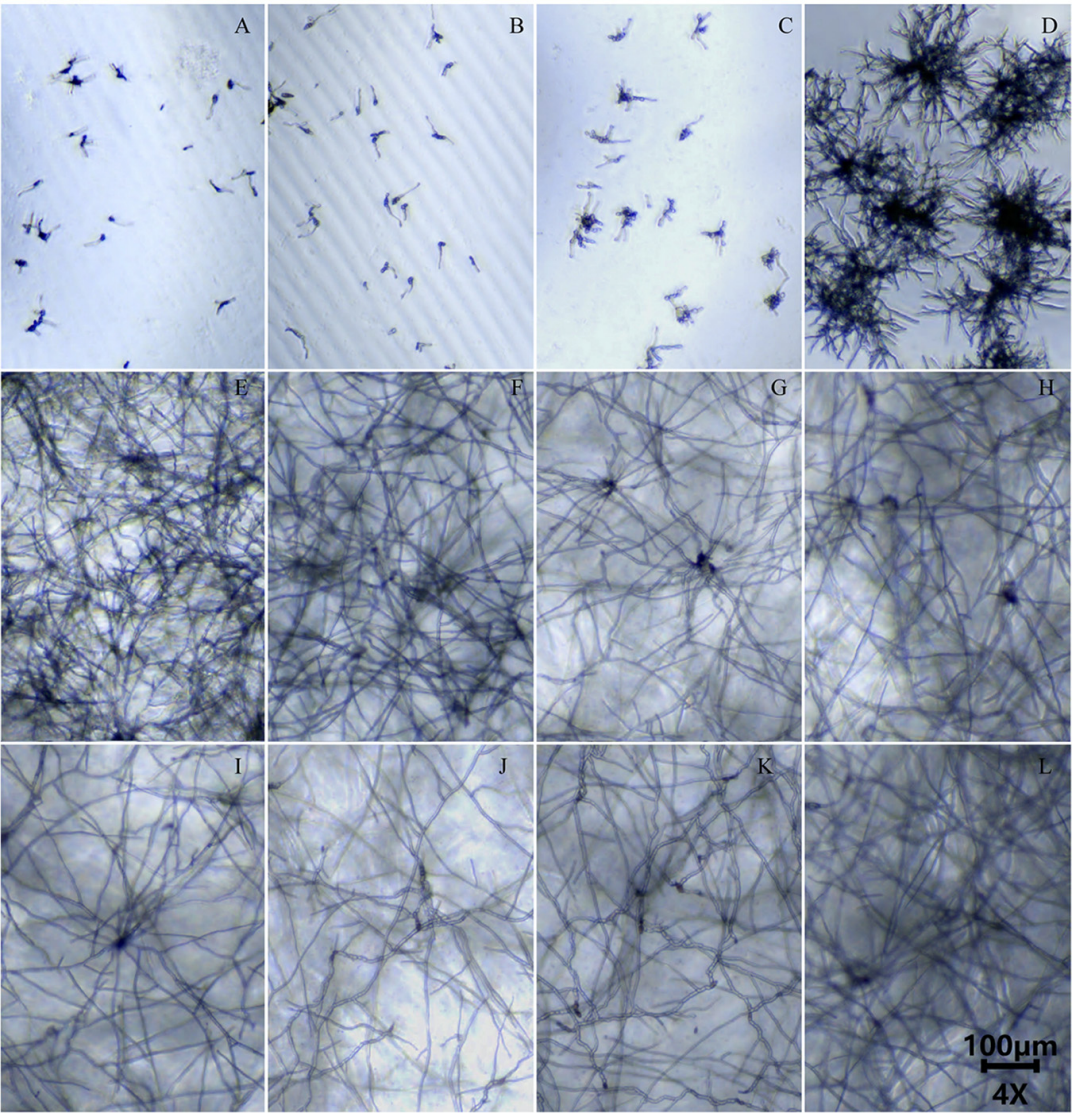
Growth of SJZ1-37 at different voriconazole concentrations. (A) 16 μg/mL, (B) 8 μg/mL, (C) 4 μg/mL, (D) 2 μg/mL, (E) 1 μg/mL, (F) 0.5 μg/mL, (G) 0.25 μg/mL, (H) 0.125 μg/mL, (I) 0.0625 μg/mL, (J) 0.03125 μg/mL, (K) 0.0156 μg/mL, (L) 0.0078 μg/mL. MIC of strain SJZ1-37 for voriconazole was 4 μg/mL.

The MIC was defined as the lowest concentration of an antimicrobial agent that causes a specified reduction in visible growth of a microorganism in an agar or broth dilution susceptibility test. At present, there is no recommended cutoff value for defining A. alternata resistance for any of the four triazoles. In our study, we examined the germination of conidia and growth of hyphae at each drug concentration for each isolate. We defined the MIC for each drug as the lowest concentration of a fungicide/drug at which conidial germination and hyphal extension of A. alternata strains were completely inhibited as determined using microscopic observations. As shown in Fig. 5 and Fig. S4 to S6, the extension of hyphae of strain SJZ1-37 was completely inhibited at 4 μg/mL for voriconazole (VOR); this concentration was determined as the MIC of strain SJZ1-37 for VOR. Our method is consistent with MIC’s previously described definition as the lowest drug concentration that inhibited any visible growth (16). The susceptibility tests of each strain were repeated three times. Two *Candida* strains, Candida parapsilosis ATCC 22019 and Candida krusei ATCC 6258, were used as references.

### Sequencing and analyses of *cyp51* gene and its upstream sequence.

Primers Altcyp51-F (5′-ATTGGATACCCTGGTCCATGC-3′) and Altcyp51-R (5′-TTAAGACCCGAAATGCGTCG-3′) were used to amplify the *cyp51* amino acid-coding gene of A. alternata. Primers Ucyp-F (5′-CAACGGCACATTTGTCAACG-3′) and Ucyp-R (5′-CAGGATAAAGGAGGCGAAGC-3′) were used to amplify the upstream sequence of *cyp51* (18). Mutations of *cyp51* gene were identified by comparing our sequences with the reference sequence of a triazole-susceptible A. alternata strain in GenBank with the accession number MN542658 (18). The potential relationship between the mutations at the *cyp51* gene and MICs among isolates for each of the four triazole drugs was assessed using the IBM SPSS statistical software version 25.0.

### Data availability.

All data supporting the conclusions of this study are presented in the article.
