# Supplementary material for: Genetic Structure and Triazole Antifungal Susceptibilities of Alternaria alternata from Greenhouses in Kunming, China
Source: Microbiol Spectr. 2022 May 12;10(3):e00382-22. doi: 10.1128/spectrum.00382-22 (PMC9241833; doi:10.1128/spectrum.00382-22)
Supplement: SUPPLEMENTAL FILE 1 — Fig. S1 to S6; Tables S1 to S5. Download spectrum.00382-22-s001.pdf, PDF file, 5.2 MB [file spectrum.00382-22-s001.pdf]

Supplementary Materials:

Title: Genetic structure and triazole antifungal susceptibilities of *Alternaria alternata* from greenhouses in Kunming, China

Authors: Guangzhu Yang [1,2,3], Sai Cui [2], Nan Ma [1], Yuansha Song [1], Jun Ma [3], Wenjing Huang [3], Ying Zhang [1]*, and Jianping Xu [1,4,*]

[1] State Key Laboratory for Conservation and Utilization of Bio-Resources in Yunnan, Key Laboratory for Southwest Microbial Diversity of the Ministry of Education, Yunnan University, Kunming 650032, P. R. China; ygzh00@mail.ynu.edu.cn (G-Z.Y.); cuisai@mail.ynu.edu.cn (S. C.); kiwi@mail.ynu.edu.cn (N.M.); yuanshasong@mail.ynu.edu.cn (Y-S.S.);

[2] School of Life Science, Yunnan University, Kunming 650032, P. R. China; ygzh@yaas.org.cn (G-Z.Y.); m001@yaas.org.cn (J.M.); YYSHWJ@ yaas.org.cn (W-J.H.)

[3] Horticultural Research Institute, Yunnan Academy of Agricultural Sciences, Kunming 650205, P. R. China; (S.C.)

[4] Department of Biology, McMaster University, Hamilton, ON L8S 4K1, Canada; jpxu@mcmaster.ca (J-P.X.)

* Correspondence: yingzhang@ynu.edu.cn (Y.Z.); jpxu@mcmaster.ca (J-P.X.)

Figure S1. Neighbor-joining tree among our isolates and with those of closely related species to *A. alternata* based on ITS sequences.

Figure S2. Bayesian consensus tree based on the concatenated DNA sequences at the ITS, *gapdh*, *tef1*, *rpb2, Alt a 1*, *endoPG* and OPA10-2 gene loci among 90 *Alternaria* strains, including nine representative strains from our greenhouse populations.

Figure S3. Neighbor-joining tree showing the relationships among 237 strains of *A. alternata*.

Figure S4: Growth of SJZ1-37 at different itraconazole concentrations.

Figure S5: Growth of SJZ1-37 at different tebuconazole concentrations.

Figure S6: Growth of SJZ1-37 at different difenoconazole concentrations.

Table S1. Summary results of AMOVA within and among populations of the *A. alternata* isolates from different greenhouses.

Table S2: Pairwise differentiations among nine greenhouse populations of *A. alternata* isolates from different greenhouses.

Table S3: Concentrations of fungicide residues in the soil sample of each greenhouse.

Table S4: Summary of Pearson correlation coefficients between the concentrations of fungicide residues in soil and the frequencies of triazole resistance.

Table S5: Pearson correlation coefficients between STR gene diversity and triazole resistance within and between *As. fumigatus* and *A. alternata.*

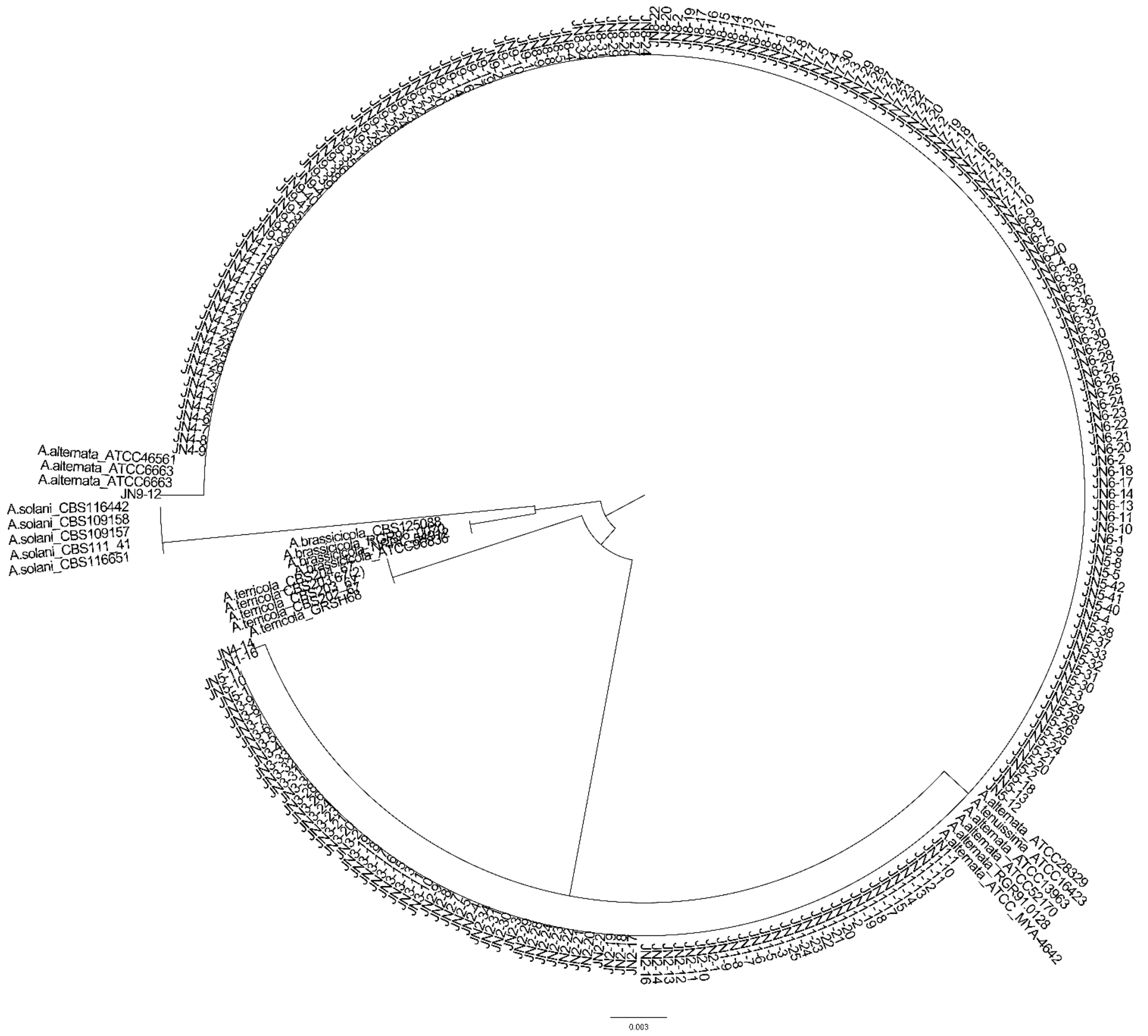

Figure S1. Neighbor-joining tree among our isolates and with those of closely related species to *A. alternata* based on ITS sequences. Our results demonstrated that all our 237 isolates belonged to *A. alternata*.

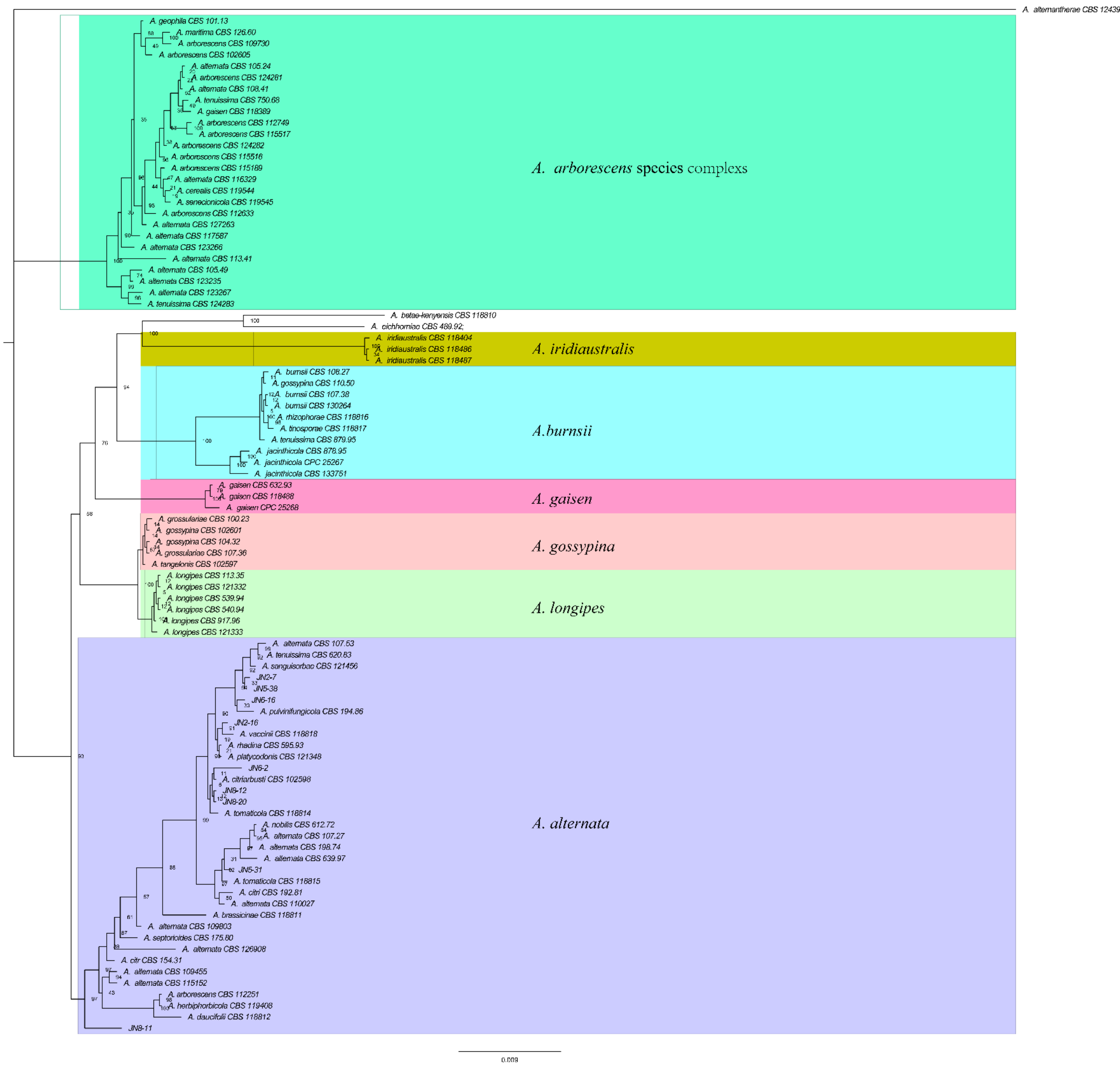

Figure S2. Bayesian consensus tree based on the concatenated DNA sequences at the ITS, *gapdh*, *tef1*, *rpb2, Alt a 1*, *endoPG* and OPA10-2 gene loci among 90 *Alternaria* strains, including nine representative strains from our greenhouse populations. The phylogenetic analysis confirmed that our strains belonged to *A. alternata* sensu stricto. The tree was rooted with *A. alternantherae* strain CBS 124392.

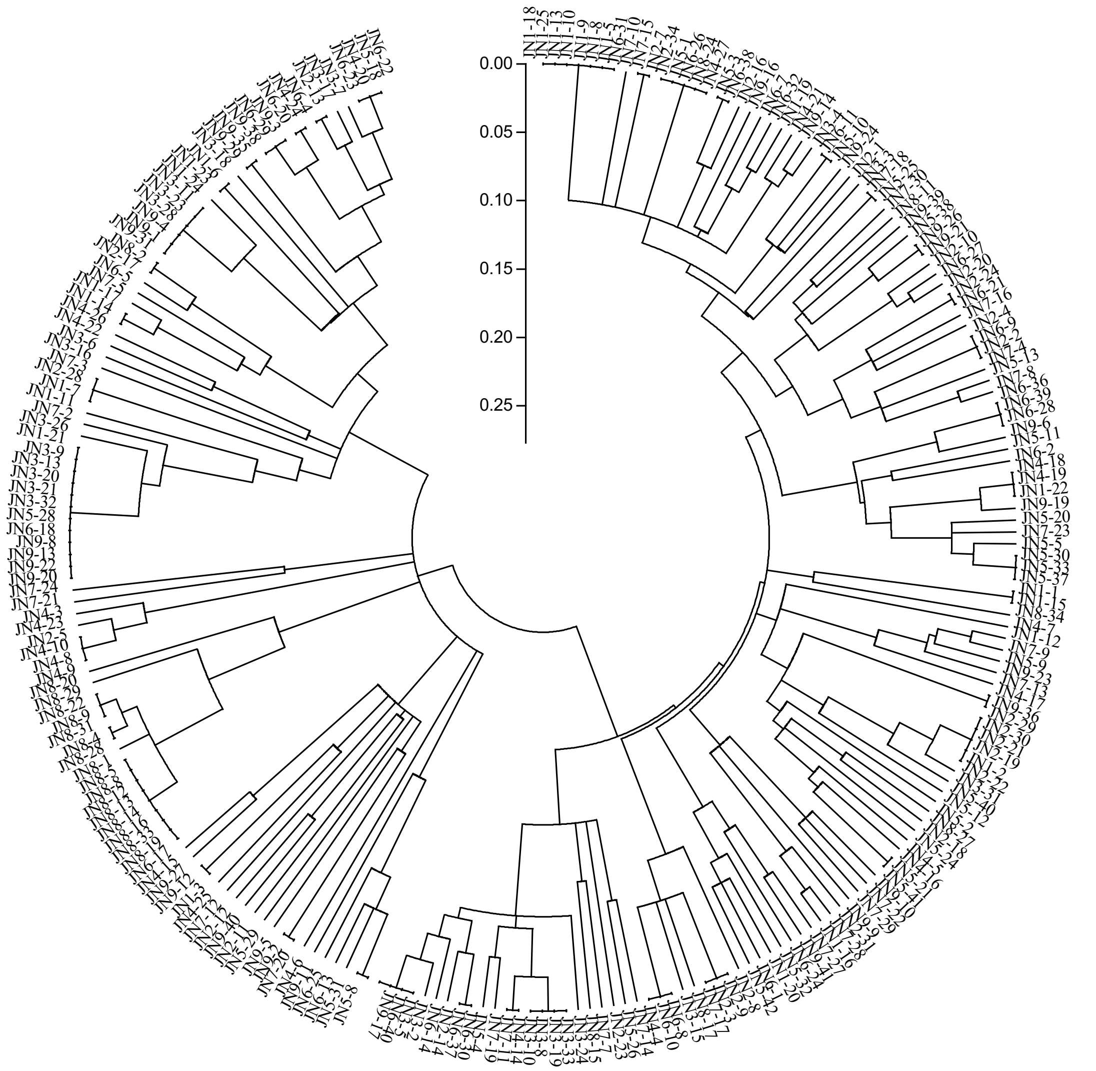

Figure S3. Neighbor-joining tree showing the relationships among 237 strains of *A. alternata.* The strain relationships were calculated based on Bruvo's distance among alleles at ten STR markers.

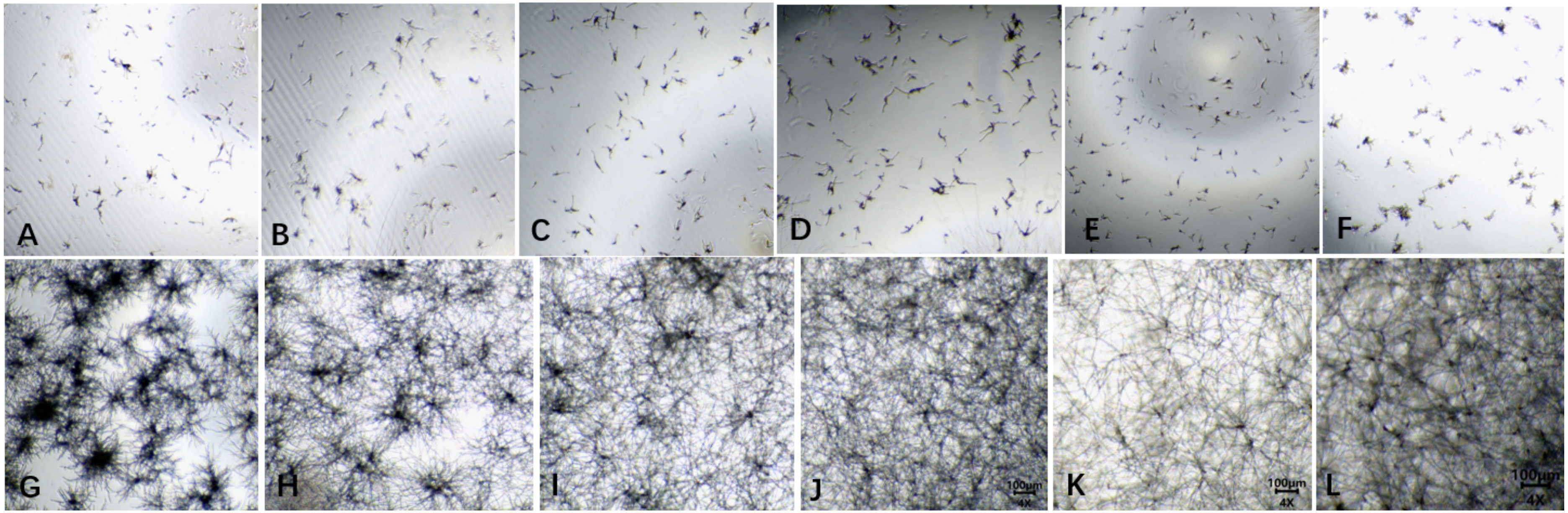

Figure S4. Growth of SJZ1-37 at different Itraconazole concentrations. A:16 μg/mL, B: 8 μg/mL, C: 4μg/mL, D:2 μg/mL, E:1 μg/mL, F: 0.5 μg/mL, G: 0.25 μg/mL, H: 0.125μg/mL, I: 0.0625 μg/mL, J: 0.03125 μg/mL, K:0.0156 μg/mL, L: 0.0078μg/mL. Minimal inhibitory concentration (MIC) of SJZ1-37 for Itraconazole was 0.5μg/mL.

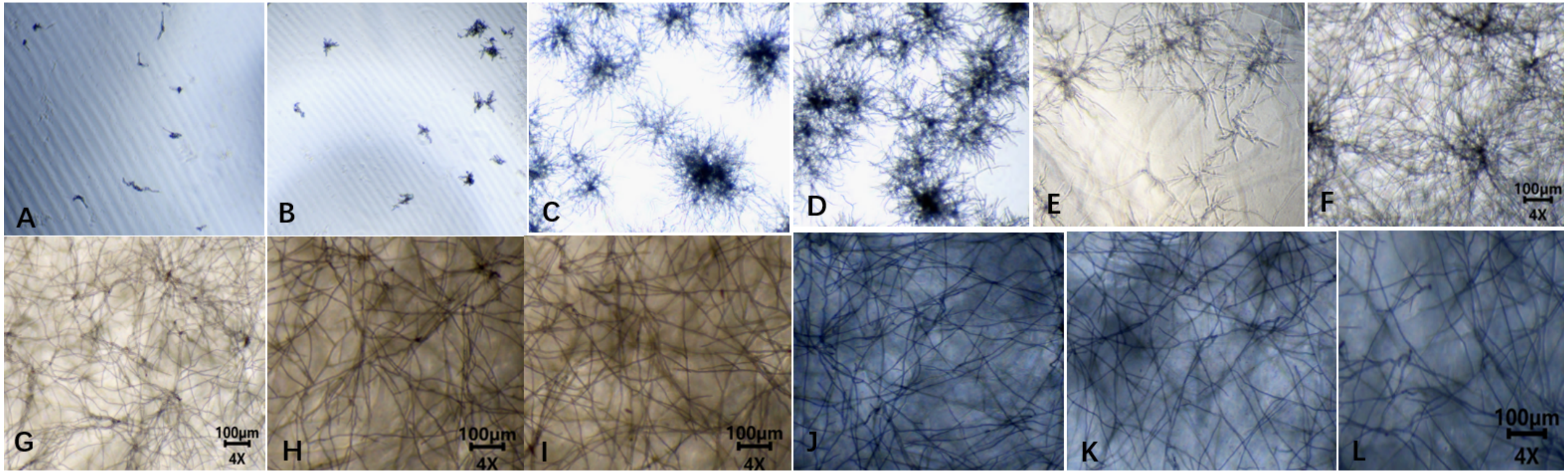

Figure S5. Growth of SJZ1-37 at different Tebuconazole concentrations. A:16 µg/mL, B: 8 µg/mL, C: 4µg/mL, D:2 µg/mL, E:1 µg/mL, F: 0.5 µg/mL, G: 0.25 µg/mL, H: 0.125µg/mL, I: 0.0625 µg/mL, J: 0.03125 µg/mL, K:0.0156 µg/mL, L: 0.0078µg/mL. MIC of SJZ1-37 for Tebuconazole was 8µg/mL.

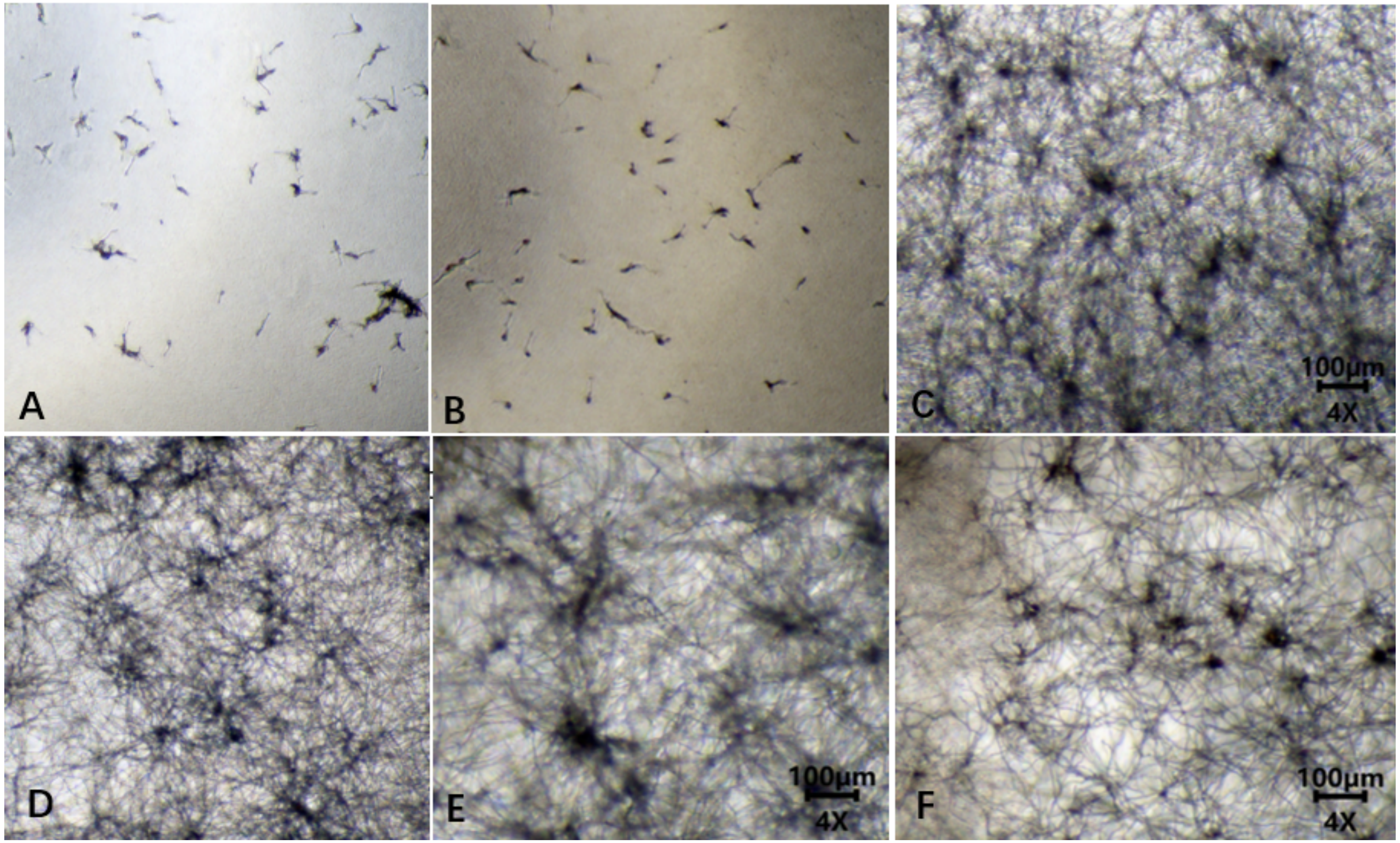

Figure S6. Growth of SJZ1-37 at different difenoconazole concentrations. A: 0.25 μg/mL, B: 0.125μg/mL, C: 0.0625 μg/mL, D: 0.03125 μg/mL, E:0.0156 μg/mL, F: 0.0078μg/mL. MIC of SJZ1-37 for difenoconazole was 0.125 μg/mL.

Table S1. Summary results of AMOVA within and among populations of the *A. alternata* isolates from different greenhouses.

Source	df	SS	MS	Est. Var.	%	AMOVA Statistics	Value	P
Among Pops	8	52.324	6.540	0.156	4%	PhiPT	0.019	0.001
Within Pops	178	592.350	3.328	3.328	96%	178	0.019	0.001
Total	186	644.674		3.484	100%			

Table S2. Pairwise differentiations among nine greenhouse populations of *A. alternata* isolates from different greenhouses. The pairwise F_{ST} values are shown below diagonal at the left bottom half of table while the *p* values are shown at the right top half of the table.

**Pop.1**	**Pop.2**	**Pop.3**	**Pop.4**	**Pop.5**	**Pop.6**	**Pop.7**	**Pop.8**	**Pop.9**	
	0.012	0.009	0.027	0.001	0.015	0.003	0.001	0.052	**Pop.1**
0.029		0.002	0.043	0.073	0.076	0.003	0.001	0.006	**Pop.2**
0.041	0.068		0.001	0.001	0.002	0.003	0.001	0.118	**Pop.3**
0.022	0.017	0.061		0.013	0.012	0.001	0.001	0.025	**Pop.4**
0.046	0.011	0.097	0.024		0.090	0.001	0.001	0.003	**Pop.5**
0.028	0.011	0.065	0.028	0.010		0.002	0.001	0.053	**Pop.6**
0.035	0.032	0.056	0.038	0.043	0.034		0.001	0.006	**Pop.7**
0.182	0.163	0.199	0.149	0.113	0.131	0.158		0.001	**Pop.8**
0.016	0.029	0.010	0.022	0.036	0.014	0.027	0.145		**Pop.9**

Table S3. Concentrations of fungicide residues in the soil sample of each greenhouse.

**pop**	Fungicide residues (μg/kg)[a]	
	Tebuconazole (μg/kg)	Difenoconazole (μg/kg)
**Pop.1**	8.82±1.01a	106.32±8.01e
**Pop.2**	4.57±0.34a	3.28±0.20a
**Pop.3**	18.94±1.37a	23.08±0.32b
**Pop.4**	8.05±0.64a	64.28±1.77c
**Pop.5**	7.54±0.73a	58.12±0.66c
**Pop.6**	345.15±26.80c	142.93±5.52f
**Pop.7**	30.89±2.66a	10.05±2.24a
**Pop.8**	56.73±4.88b	79.20±7.58d
**Pop.9**	2.51±0.39a	3.86±0.09a

[a] Different letters indicate significant differences at $P < 0.05$ in the same column.

Table S4. Summary of Pearson correlation coefficients between the concentrations of fungicide residues in soil and the frequencies of triazole resistance.

Analyzed pairs of quantitative traits	Correlation Results by SPSS	
	Correlation coefficient	P value
Concentration of TEB and frequency of TEB resistance	0.303	0.429
Concentration of TEB and frequency of DIF resistance	-0.136	0.728
Concentration of TEB and frequency of ITR resistance	0.406	0.278
Concentration of TEB and frequency of VOR resistance	0.149	0.703
Concentration of TEB and gene diversity	-0.173	0.657
Concentration of DIF and frequency of TEB resistance	-0.017	0.964
Concentration of DIF and frequency of DIF resistance	-0.133	0.734
Concentration of DIF and frequency of ITR resistance	0.153	0.693
Concentration of DIF and frequency of VOR resistance	-0.169	0.664
Concentration of DIF and gene diversity	-0.246	0.523

Table S5. Pearson correlation coefficients between STR gene diversity and triazole resistance within and between *As. fumigatus* and *A. alternata.*

		As. fumigatus				*A. alternata*				
		Gene diversity	ITR resistance	VOR resistance	TEB resistance	Gene diversity	ITR resistance	VOR resistance	TEB resistance	DIF resistance
As. fumigatus	Gene diversity		0.975	0.201	0.197	0.603	--	--	--	--
	ITR resistance	-0.012		0.005	0.007	--	0.120	--	--	--
	VOR resistance	-0.470	-0.184**		0.028	--	--	0.947	--	--
	TEB resistance	-0.474	-0.177**	0.144*		--	--	--	0.651	--
A. alternata	Gene diversity	-0.201	--	--	--		0.134	0.229	0.526	0.576
	ITR resistance	--	0.556	--	--	-0.540		0.035	0.261	0.199
	VOR resistance	--	--	-0.026	--	-0.446	0.701*		0.000	0.003
	TEB resistance	--	--	--	-0.176	0.245	0.084	0.589***		0.158
	DIF resistance	--	--	--	--	0.221	0.467	0.216**	0.105	

Pearson correlation coefficients are shown below diagonal at bottom left portion of table. *p* values are shown above diagonal at top right portion of table.
